# Plasma-derived extracellular vesicles miR-335–5p as potential diagnostic biomarkers for fusion-positive rhabdomyosarcoma

**DOI:** 10.1186/s13046-024-03197-3

**Published:** 2024-10-09

**Authors:** Virginia Di Paolo, Alessandro Paolini, Angela Galardi, Patrizia Gasparini, Loris De Cecco, Marta Colletti, Silvia Lampis, Salvatore Raieli, Cristiano De Stefanis, Evelina Miele, Ida Russo, Valentina Di Ruscio, Michela Casanova, Rita Alaggio, Andrea Masotti, Giuseppe Maria Milano, Franco Locatelli, Angela Di Giannatale

**Affiliations:** 1 Present Address: Hematology/Oncology and Cell and Gene Therapy Unit, Bambino Gesù Children’s Hospital, IRCCS, Rome, Italy; 2 Multifactorial and Complex Phenotype Research Area, Bambino Gesù Children’s Hospital-IRCCS, Rome, Italy; 3 Tumor Genomics Unit, Department of Research, Fondazione IRCCS Istituto Nazionale Dei Tumori, Milan, Italy; 4 Molecular Mechanisms Unit, Department of Research, Fondazione IRCCS Istituto Nazionale Dei Tumori, Milan, Italy; 5 Oncodesign SA, Dijon, 21079 France; 6 Research Laboratories, Bambino Gesù Children’s Hospital, IRCCS, Rome, Italy; 7 Pediatric Oncology Unit, Fondazione IRCCS Istituto Nazionale Dei Tumori, Milan, Italy; 8 Pathology Unit and Predictive Molecular Pathology Unit, Bambino Gesù Children’s Hospital, IRCCS, Rome, Italy; 9 Department of Life Sciences and Public Health, Catholic University of the Sacred Heart, Rome, Italy

**Keywords:** Rhabdomyosarcoma, MicroRNA, Extracellular vesicles, Liquid biopsy

## Abstract

**Background:**

Rhabdomyosarcoma (RMS) is the most common pediatric soft tissue sarcoma, with embryonal (ERMS) and alveolar (ARMS) representing the two most common histological subtypes. ARMS shows poor prognosis, being often metastatic at diagnosis. Thus, the discovery of novel biomarkers predictive of tumor aggressiveness represents one of the most important challenges to overcome and may help the development of tailored therapies. In the last years, miRNAs carried in extracellular vesicles (EVs), small vesicles of endocytic origin, have emerged as ideal candidate biomarkers due to their stability in plasma and their tissue specificity.

**Methods:**

EVs miRNAs were isolated from plasma of 21 patients affected by RMS and 13 healthy childrens (HC). We performed a miRNA profile using the Serum/Plasma Focus microRNA PCR panels (Qiagen), and RT-qPCR for validation analysis. Statistically significant (*p* < 0.05) miRNAs were obtained by ANOVA test.

**Results:**

We identified nine EVs miRNAs (miR-483-5p, miR-132-3p, miR-766-3p, miR-454-3p miR-197-3p, miR-335-3p, miR-17-5p, miR-486-5p and miR-484) highly upregulated in RMS patients compared to HCs. Interestingly, 4 miRNAs (miR-335-5p, miR-17-5p, miR-486-5p and miR-484) were significantly upregulated in ARMS samples compared to ERMS. In the validation analysis performed in a larger group of patients only three miRNAs (miR-483-5p, miR-335-5p and miR-484) were differentially significantly expressed in RMS patients compared to HC. Among these, mir-335-5p was significant also when compared ARMS to ERMS patients. MiR-335-5p was upregulated in RMS tumor tissues respect to normal tissues (*p* = 0.00202) and upregulated significantly between ARMS and ERMS (*p* = 0.04). Furthermore, the miRNA expression correlated with the Intergroup Rhabdomyosarcoma Study (IRS) grouping system, (*p* = 0.0234), and survival (OS, *p* = 0.044; PFS, *p* = 0.025). By performing in situ hybridization, we observed that miR-335-5p signal was exclusively in the cytoplasm of cancer cells.

**Conclusion:**

We identified miR-335-5p as significantly upregulated in plasma derived EVs and tumor tissue of patients affected by ARMS. Its expression correlates to stage and survival in patients. Future studies are needed to validate miR-335-5p as prognostic biomarker and to deeply elucidate its biological role.

**Supplementary Information:**

The online version contains supplementary material available at 10.1186/s13046-024-03197-3.

## Background

Rhabdomyosarcoma (RMS) is the most common type of soft tissue sarcoma in children and young adults, accounting for up to 3–4% of childhood cancer and approximately 50% of all sarcomas [1, 2]. Embryonal (ERMS) and alveolar (ARMS) RMS represent the two most common histological subtypes. ARMS is associated with two chromosomal translocations, t(2;13) (q35;q14) and t(1;13) (p36;q14) resulting respectively in the PAX3–FOXO1 and PAX7–FOXO1 fusion proteins [3]. Various studies have demonstrated that PAX3-7-FOXO1 fusion oncoprotein enhances RMS growth and metastasis by targeting genes involved in proliferation, migration and invasion [4]. Patients affected by ARMS shows a poor prognosis, being often metastatic at diagnosis.

Although, during the last three decades, the use of combination therapies has substantially improved the prognosis of localized RMS, the clinical outcomes for children with metastatic RMS remains very poor even with a multimodal approach, with a 5-year event free survival (EFS) and overall survival (OS) of 17.3% and 21.3% respectively [5, 6]. Thus, the discovery of novel biomarkers predictive of tumor aggressiveness may help the development of tailored therapies and represents one of the most important objectives to achieve in this disease.

Liquid biopsy allows to identify tumor secreted factor circulating in the body fluids, such as plasma or serum, with the advantage of being minimally invasive and reflecting tumor burden in patients [7]. This approach enables the detection of circulating tumor cells, cell-free DNA (cfDNA), circulating microRNAs (miRNAs), proteins and tumor cell-derived extracellular vesicles (EVs) [8]. EVs are cell membrane-derived nanovesicles (30 nm -10 μm) released by eukaryotic cells and abundantly by aggressive tumor cells, carrying lipids, soluble and transmembrane proteins, mRNAs, miRNAs and double-stranded DNA. EVs play a role in intercellular communication by transferring molecules to the surrounding cells and may have significant contribution to tumor progression [9, 10]. Consequently theyare promising candidates as specific cancer biomarkers. MiRNAs within extracellular vesicles (EVs-miRNAs) are particularly stable as they are protected from RNAses, providing an enriched and ideal source for tumor biomarkers detection [11, 12]. Tumor-secreted EVs-miRNAs can be transferred to target cells, influencing their gene expression and impacting tumor biology [13–15]. In RMS, several functional studies have demonstrated that different miRNAs could act both as tumor suppressor and oncomiRs regulating cancer cell proliferation, invasion, and apoptosis [16–23]*.* The study of miRNAs, particularly those encapsulated in EVs, provides unique insights due to their stability, resistance to degradation. Compared to studying mRNA derived from cfRNA, miRNAs offer complementary information as they regulate gene expression and could help to identify the mechanism involved in RMS aggressiveness. Notably, it has been observed that EVs derived from RMS cell lines are enriched in miRNAs, which are implicated in inducing angiogenesis, tumor growth and metastasis [24].

Herein, we report an exploratory study on EVs-miRNAs derived from the plasma of RMS patients, with the purpose of identifying predictive diagnostic and prognostic biomarkers. We found that among the significantly dysregulated miRNAs, miR-335-5p correlates with ARMS subtype and with prognosis in RMS patients. To our knowledge, this is the first study reporting a potential interest of miR-335-5p as novel biomarker in RMS patients.

## Materials and methods

### Patients and sample collection

Plasma was collected from 21 RMS patients at diagnosis and 13 healthy children (HC) at Pediatric Haematology/Oncology and Cell and Gene Therapy Department, Bambino Gesù Children’s Hospital. Among the RMS patients 9 were diagnosed with fusion positive ARMS and 12 with ERMS, 8 were females and 13 males; their median age was 49 months (range: 5–188 months). Among HC, 8 were males and 5 females, their median age was 74,9 months (range: 6–207 months). Patients’ clinical information is shown in Supplementary Table 1. Written informed consent was signed by all parents and the study was approved by our Institutional Ethics Committee (protocol number 1189_OPBG_2016).

Whole blood was collected in EDTA tubes (BD Vacutainer, Reading, UK) and processed within 2 h. The samples were first centrifugated at 500 × g for 10 min, and then supernatants were collected and centrifuged at 3000 × g and then at 12,000 × g for 20 min each. All the centrifugation steps were performed at 4 degrees. The plasma was collected and stored at -80◦C until EVs isolation.

### Isolation of extracellular vesicles from plasma

EVs isolation from plasma was performed using the commercial kit miRCURY™ Exosome isolation kit-serum and plasma (Qiagen) according to the manufacturer’s protocol. Briefly, 3 UI of Thrombin was added to 0.6 ml of plasma and incubated for 5 min at room temperature (RT) and centrifuged for 5 min at 10,000 × g. An amount of 0.5 ml of supernatant was collected, 200 μl of precipitation buffer A was added, resuspended by vortexing for 5 s to mix and incubated for 60 min at 4 °C. After incubation, samples were centrifuged for 5 min at 500 g at RT and the supernatants were removed and discarded. Pellets were re-suspended by vortex in 270 μl resuspension buffer. The isolated EVs were characterized following the recommendations of “Minimal Information for Studies of Extracellular Vesicles” (MISEV) 2023, (Supplementary Methods) [25]. Transmission Electron Microscopy (TEM) confirmed the presence of EVs with homogeneous morphology, occasionally clustered, with a size ranging from 30 to 200 nm (Supplementary Fig. 1A). NanoSight analysis showed a mean vesicle diameter ranging from 105 to 146 nm (Supplementary Fig. 1B). Western Blot revealed an enrichment of the EVs-specific protein Tumor Susceptibility Gene 101 (TSG101), CD9, and CD63 in nanovesicles samples compared to Hela cells lysate; furthermore, the absence of endoplasmic reticulum protein Calnexin demonstrate that no cell debris were present in our preparation’s lysate (Supplementary Fig. 1C). The purified EVs samples were then processed for RNA extraction.

### RNA isolation from plasma extracellular vesicles

RNA from plasma EVs was isolated using miRCURY RNA isolation kit-biofluids (Qiagen) according to the manufacturer’s protocol. Briefly, 300 μl of resuspended EVs were mixed with 90 μl Lysis solution biofluids (BF), vortexed for 5 s and incubated for 10 min at RT. 1 μl of RNA spike-in template mixture (miRCURY LNA™ Universal RT microRNA PCR, RNA spike-in kit) was added to each sample for downstream PCR analysis. Then, 30 μl Protein precipitation solution BF was added to samples and vortexed, incubated for 1 min at RT and centrifuged for 3 min at 11,000 g. The supernatants, after addition of 400 μl isopropanol, were vortexed for 5 s and then loaded in miRNA mini spin column BF. Columns were incubated for 2 min at RT, centrifuged for 30 s at 10,000 g, washed with Wash solution 1 BF and twice with Wash solution BF 2. Columns were centrifuged for 2 min at 11,000 g to dry membranes and RNA was eluted adding 30 μl RNase free H_2_O directly onto the membrane of the spin columns BF. Columns were incubated for 1 min at RT and then centrifuged for 1 min at 11,000 g. The purified RNA samples were stored at -80 °C.

### qPCR assessment of extracellular vesicles miRNAs

Total RNA extracted from plasma exosomes was mixed with two artificial RNAs (RNA spike-ins as RT controls) and the final mixture (10 μl) was reverse transcribed at 42 °C for 60 min using the miRCURY LNA™ Universal RT cDNA Synthesis Kit (Qiagen) following the manufacturer’s instruction. The expression level of plasma EVs-miRNAs was evaluated by Serum/Plasma Focus microRNA PCR panels (Qiagen). The amplification curves were filtered (Ct < 36), imported into the GenEx software (ver.5, Qiagen) and normalized by global mean. The expression level (fold change [FC]) was calculated by taking the mean of individual Cq values for each group (HC, ERMS and ARMS patients). To validate the significant EVs-miRNAs in the plasma of an independent cohort of patients, we individually assayed mature miR-486-5p (cat.no. 339306-YP00204001), miR-17-5p (cat.no. 339306-YP02119304), miR-197-3p (cat.no. 339306-YP00204380), miR-483-5p (cat.no. 339306-YP00205693), miR-766-3p (cat.no. 339306-YP00204499), hsa-miR-132-3p (cat.no. 339306-YP00206035), hsa-miR-454-3p (cat.no. 339306-YP00205663), hsa-miR-484 (cat.no. 339306-YP00205636) and hsa-miR-335-5p (cat.no. 339306-YP02119293), by employing two endogenous miRNAs, namely miR-23a-3p (cat.no. 339306-YP00204772) and miR-320a (cat.no. 339306-YP00206042) that were selected by running Genorm and NormFinder analysis tools. QuantStudio 12 K Flex Real-Time PCR System (Thermo Fisher Scientific, Waltham, MA, USA) was employed for all the qPCR quantifications and the fold change was calculated by the 2^−ΔΔCt^ method [26]. At least two independent amplifications were performed for each probe on triplicate samples. The raw Cq values from amplification curves (Serum/Plasma plates) were normalized by global mean using the GenEx qPCR analysis software (Exiqon ver 5), individual assays were normalized by taking miR-23a-3p and miR-320a as endogenous controls. Statistically significant (*p* < 0.05) miRNAs were obtained by ANOVA test (ERMS patients and ARMS patients versus controls). MiRNAs with a FC lower than -2 (FC < -2) and greater than 2 (FC > 2) in RMS patients and with a *p-*value lower than 0.05 compared to controls were considered highly dysregulated and retained for further bioinformatics analysis.

### Bioinformatics analysis of Gene Expression Omnibus (GEO) dataset

A survey on the Gene Expression Omnibus (GEO, https://www.ncbi.nlm.nih.gov/gds) repository was made to identify the publicly available miRNA expression datasets associated with RMS patients. The miRNA microarray-based expression data matrix from a cohort of 49 RMS Formalin-fixed paraffin-embedded tissues (primary not pre-treated tumors) collected at Fondazione IRCCS Istituto Nazionale dei Tumori (Milan, Italy) was retrieved for further analysis (ID: GSE135518). In this dataset, miRNA profile was performed using a SurePrint G3 Human miRNA r21 microarrays (Agilent) designed on miRBase 21.0 (miRNA). GSE135518 includes 27 pediatric RMS (0–14 years) and 22 AYA RMS (15- + 30 years)] as well as 13 normal tissue counterparts (CTRL) [27]. Primary data were collected using Agilent’s Feature Extraction software v10.7 (Agilent Technologies), background corrected, and quantile normalized using Bioconductor limma implementation in R. Differentially expressed miRNAs in the tissue sample between ARMS, ERMS and CTRL were identified imposing log2|FC|> 2 and adjusted *p* < 0.05 by using the GEO2R bioinformatics tool. Expression of miR-335-5p was retrieved from the data matrix and median expression was used to stratify patients for Kaplan–Meier analysis having overall survival as clinical endpoint. Patients’ clinical information’s are shown in Supplementary Table 2.

### Bioinformatics target prediction of miR-335-5p and gene ontology

Target prediction of the miR-335-5p, was carried out by already reported procedure [28] that integrates the predictions of three different algorithms (i.e., TargetScan, MiRanda, and PITA). The list of target genes predicted in all databases were used for bioinformatics analysis. Gene Ontology (GO), KEGG pathway enrichment analysis, and annotation by DAVID bioinformatics tool [29] were performed, to determine the biological processes and signaling pathways in which the predicted targets of miR-335-5p were involved.

### In situ hybridization (ISH)

Tumor sample paraffin embedded from 16 patients (10 ERMS and 6 ARMS) were obtained from the archives of Operative Unit of Pathology at Bambino Gesù Children’s Hospital. Formalin-fixed paraffin-embedded tumor samples were cut in RNAse-free environment at 5 µm thick, mounted on positive-charged slides. MiRNA in situ hybridization was performed as previously described [15]. Slides were analyzed by light microscopy with (Eclipse E600, Nikon). Each slide was scored by 2 independent, qualified observers, blinded on patient’s clinical information’s. miR-335-5p expression was semi-quantitatively evaluated based on staining intensity and distribution using a total score as follows: intensity score × proportional score. The intensity score (IS) was predefined as follows: 0, negative; 1, weak; 2, moderate and 3, strong. The proportional score (PS) was defined as follows: 0, negative; 1, < 10%; 2, 10%–50%; 3, > 50% positive cells [30].

## Results

### Profiling of extracellular vesicles miRNAs in plasma of RMS patients

**Fig. 1:**
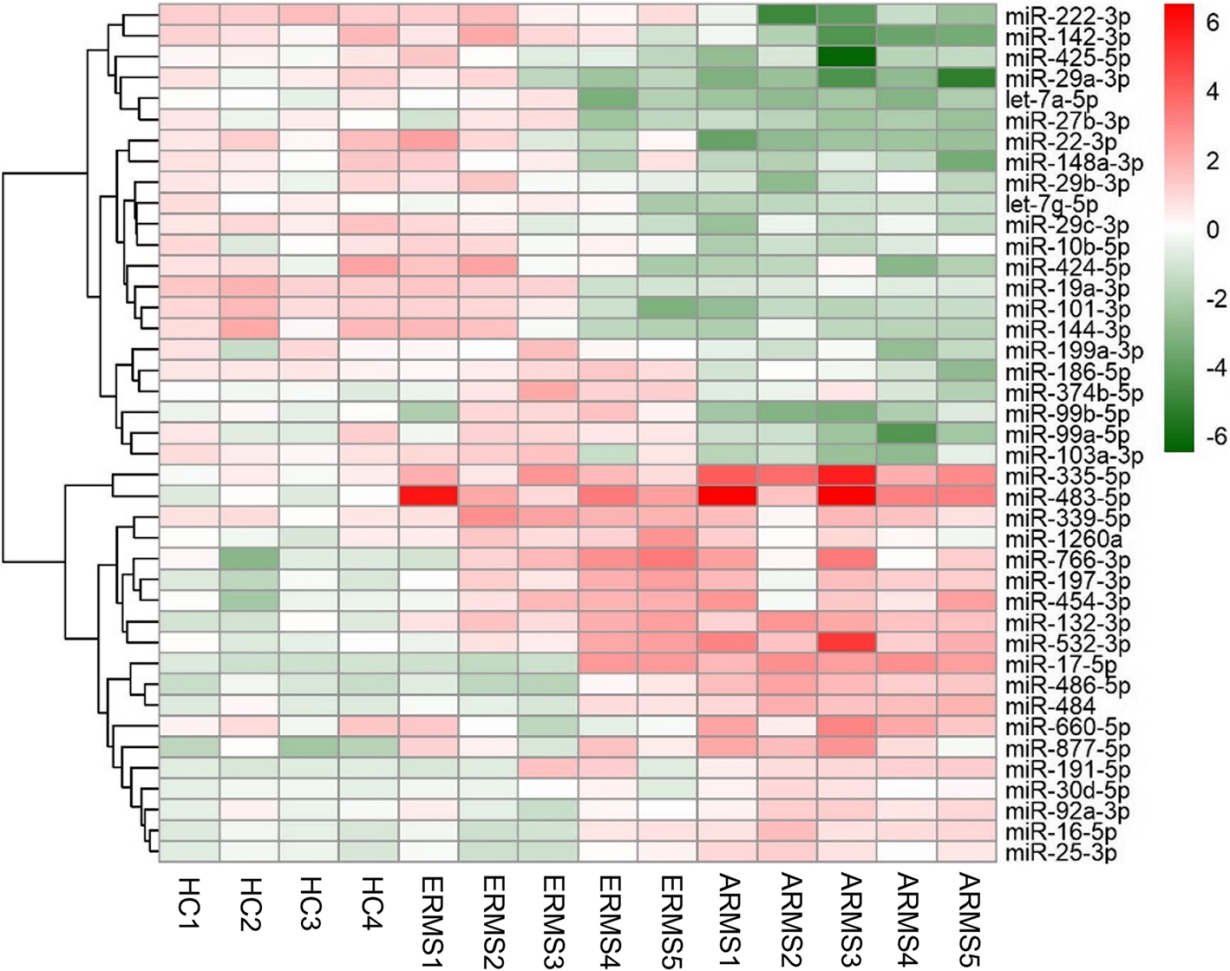
Heatmap of the 41 statistically dysregulated EVs-miRNAs in ERMS and ARMS patients compared to HC. Upregulated and downregulated miRNAs are represented in red and green, respectively, *P-*value < 0.05

We first analyzed EVs-miRNAs differentially expressed in the comparison between 10 RMS patients’ group (5 ARMS and 5 ERMS) and 4 HCs. A total of (171) miRNAs were expressed in RMS patients and (180) in HC. Among commonly expressed miRNAs, we focused on the EVs-miRNAs expressed in the 80% of RMS samples and HCs (116). A total of 41 miRNAs were significantly (p < 0.05) up or down regulated in one of the two groups (i.e., ERMS and ARMS), (Fig. 1, Supplementary Table 3).

Nine miRNAs (miR-483-5p, miR-132-3p, miR-766-3p, miR-454-3p miR-197-3p, miR-335-3p, miR-17-5p, miR-486-5p and miR-484) were highly upregulated in RMS patients compared to HCs, whereas no significant downregulated miRNAs were observed (Table 1).

**Table 1:** Highly dysregulated and statistically significant miRNAs (-2 > FC > 2; *p-*value < 0.05) detected in RMS patients compared to HCs, in ERMS and ARMS patients compared to HCs and in ARMS patients compared to ERMS patients

***ID***	***Relative quantity (FC* ± *St. Dev)***				***P****-Value*			
	ERMS	ARMS	RMS	ANOVA *p-*value	RMS vs HC	ERMS vs HC	ARMS vs HC	ARMS vs ERMS
hsa-miR-483-5p	7.72 ± 3.62	18.34 ± 4.59	11.9 ± 4.09	**0.007**	**0,002**	**0.016**	**0.002**	0.284
hsa-miR-132-3p	2.83 ± 1.61	3.45 ± 1.57	3.12 ± 1.57	** < 0.001**	**0,000**	**0.000**	** < 0.001**	0.495
hsa-miR-766-3p	2.9 ± 3.28	2.75 ± 2.72	2.83 ± 2.82	**0.046**	**0,011**	**0.026**	**0.030**	0.938
hsa-miR-454-3p	2.3 ± 1.99	2.6 ± 2.34	2.45 ± 2.08	**0.030**	**0,007**	**0.023**	**0.015**	0.808
hsa-miR-197-3p	2.4 ± 2.03	2.13 ± 1.79	2.26 ± 1.85	**0.006**	**0,001**	**0.003**	**0.005**	0.752
hsa-miR-335-3p	3.01 ± 1.812	12.87 ± 2.62	6.23 ± 2.93	**0.001**	**0,009**	0.053	** < 0.001**	**0.007**
hsa-miR-17-5p	1.17 ± 4.39	5.51 ± 1.38	2.54 ± 3.66	**0.006**	**0,026**	0.161	**0.002**	**0.022**
hsa-miR-486-5p	-1.54 ± 2.07	3.2 ± 1.33	1.44 ± 2.69	< 0.001	0,062	0.447	** < 0.001**	** < 0.001**
hsa-miR-484	-1.01 ± 1.75	2.98 ± 1.31	1.72 ± 2.04	**0.001**	**0,031**	0.205	** < 0.001**	**0.002**

Performing the analysis by histology, 5 miRNAs (miR-483-5p, miR-132-3p, miR-766-3p, miR-454-3p and miR-197-3p) were significantly upregulated in both ERMS and ARMS patients compared to HCs. Interestingly, 4 miRNAs (miR-335-5p, miR-17-5p, miR-486-5p and miR-484) were significantly upregulated in ARMS samples compared to ERMS patients (Table 1).

### Validation of selected extracellular vesicles miRNAs from plasma by RT-qPCR

**Fig. 2:**
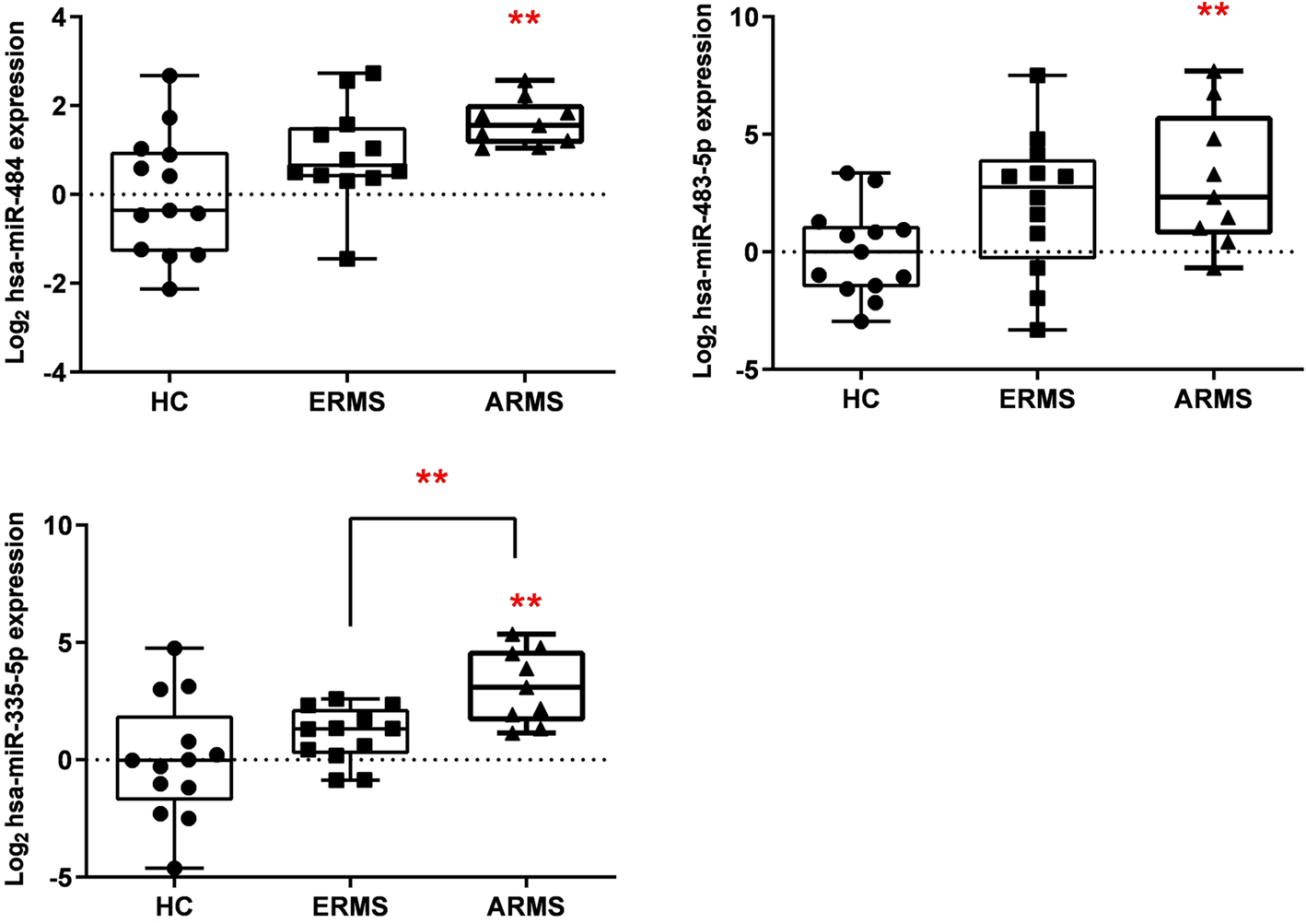
Significant EVs-miRNAs expression in the validation analysis evaluated by RT-qPCR. ** *p* < 0,01 (miRNA-484, miRNA-483-5p, miRNA-335-5p)

In order to validate the results obtained in our previous discovery analysis (Table 1), the expression level of the nine EVs-miRNAs (miR-483-5p, miR-132-3p, miR-766-3p, miR-454-3p and miR-197-3p, miR-335-5p, miR-17-5p, miR-486-5p and miR-484), found to be significantly upregulated in RMS were validated in additional samples by using real-time qPCR. In total, 12 ERMS, 9 ARMS and 13 HC were used in the validation analysis (Supplementary Table 1). The results, obtained by ANOVA test, showed that only miR-483-5p (*p* = 0.029), miR-484 (*p* = 0.007) and miR-335-5p (*p* = 0.003) resulted statistically significant between groups (Fig. 2). Interestingly, the three miRNAs were significantly upregulated in ARMS patients compared to HC and only mir-335-5p was significantly upregulated also compared to ERMS (Fig. 2). This suggest that miR-335-5p could be specifically related to ARMS histotype.

## Time-course analysis of miR-335-5p in two patients during treatment

We also analyzed EVs miR-335-5p expression during different treatment timepoints in two patients (ARMS 1 and ERMS 2), (Supplementary Fig. 2).

ARMS 1 was a 9 months old boy diagnosed with a parameningeal ARMS with bone and lymph nodes metastasis [2]. At the end of maintenance treatment with vinorelbine and cyclophosphamide, he presented a local relapse of disease. He received second line of treatment with vincristine, irinotecan and temodal [31]. The patients died after 2 cycles of VIT for further progression disease (PD). The expression of EVs miR-335-5p reduced during maintenance treatment but increased close to the end of maintenance and at the relapse.

ERMS 2 was a 8 years old girl diagnosed with a localized parameningeal ERMS (size more than 5 cm diameter, IRS III, N1, M0). She received chemotherapy according to EpSSG RMS 05 protocol, HR group. After the first 3 cycles of chemotherapy, she showed a partial response. She continued scheduled chemotherapy, and she received radiotherapy on the tumor and lymph nodes. For local PD, she was treated with a second line chemotherapy with vincristine, irinotecan and pazopanib [32]. The expression of EVs miR-335-5p reduced after the third cycle of chemotherapy, but increased again after the 4th cycle and at the diagnosis of local PD. She is still alive after 6 years of treatment stop.

### Prediction of target genes and hub genes of Kegg pathway network of miR-335-5p

**Fig. 3:**
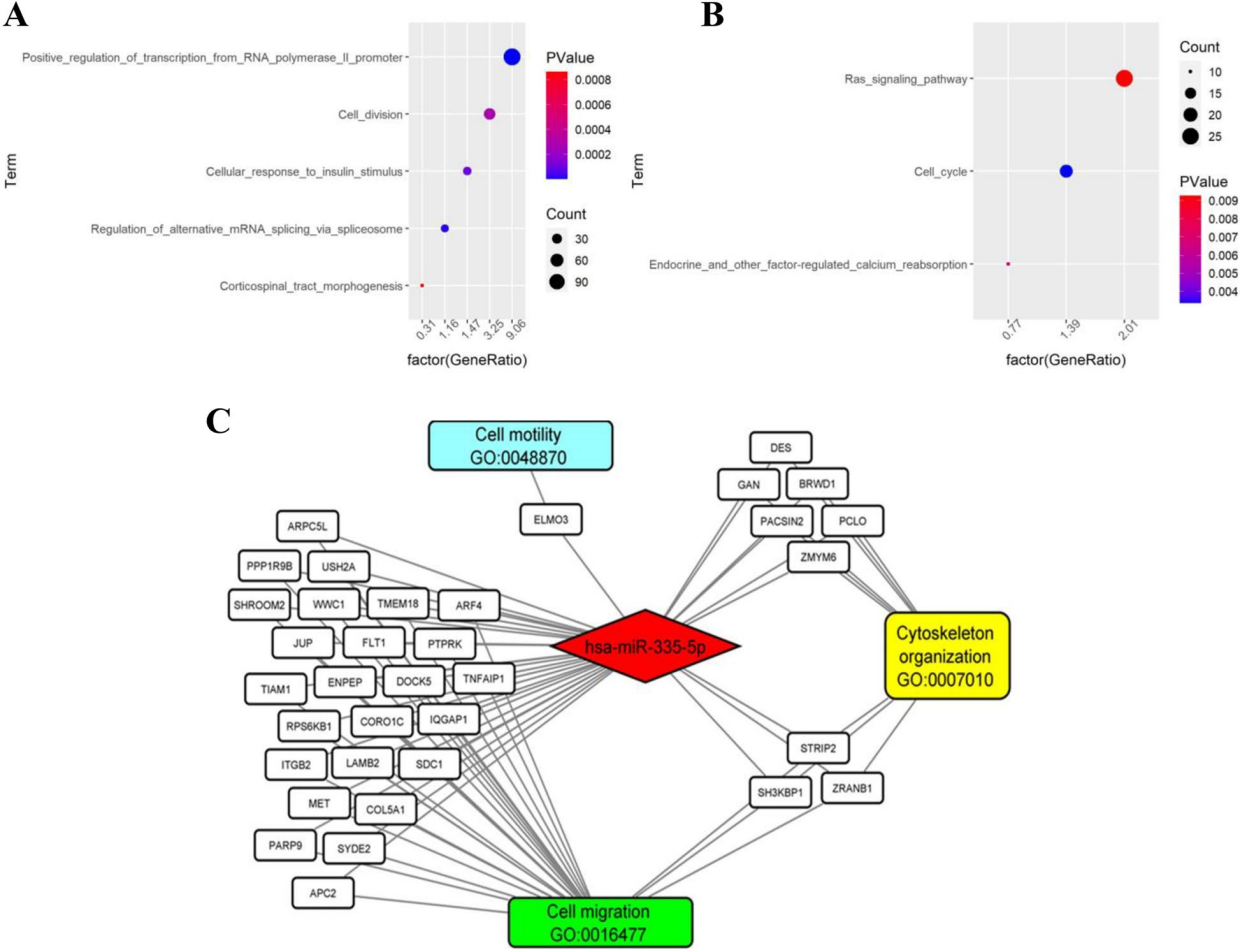
Bioinformatic analysis of predicted targets of miR-335-5p. **A** GO enrichment analysis; **B** KEGG Pathways; **C** Cytoscape interaction network

By following our bioinformatics approach, we obtained a list of 1292 target genes predicted for miR-335-5p. By GO enrichment analysis (http://amigo.geneontology.org) we found that the most enriched items with a *p-*value < 0.001 are: positive regulation of transcription from RNA polymerase II promoter (GO:0045944); cell division (GO:0051301); cellular response to insulin stimulus (GO:0032869); regulation of alternative mRNA splicing, via spliceosome (GO:0000381); corticospinal tract morphogenesis (GO:0021957), (Fig. 3A). The KEGG pathways analysis showed an enrichment in target genes with a *p-*value < 0.01 implicated in: Ras signaling pathway (hsa04014) [33]; Cell cycle (hsa04110); Endocrine and other factor-regulated calcium reabsorption (hsa04961), (Fig. 3B).

Finally, we carried out a bioinformatic analysis to identify the target genes of miR-335-5p involved in biological processes that contribute to metastatic behavior such as cell migration (GO:0016477), cell motility (GO:0048870), and cytoskeleton organization (GO0007010). In GO biological processes, 257 genes belong to cell migration, 31 to cell motility, 134 to cytoskeleton organization and 35 genes were in common in all of the three pathways. We extracted the intersection of these lists and the connections between miR-335-5p, and its target genes were visualized in Fig. 3C as an interaction network (by Cytoscape).

### MiR-335-5p is upregulated in RMS tissues

**Fig. 4:**
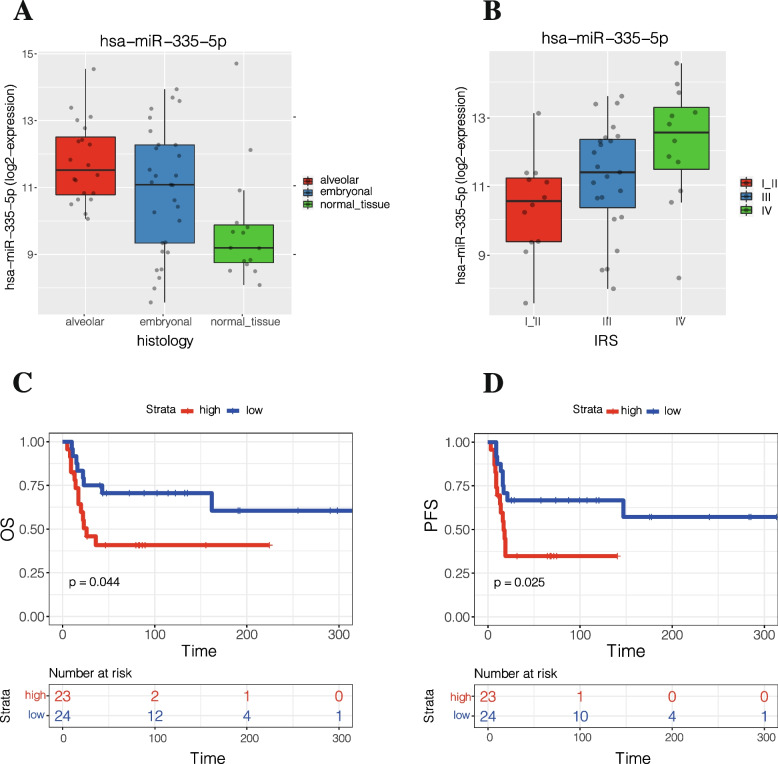
Bioinformatics analysis of expression of miR-335-5p, from GSE135518 database in RMS tissues compared to normal tissue. **A** Comparison between miR-335-5p expression and histology **B** Comparison between miR-335-5p and IRS; **C** Correlation between miR-335-5p and OS and (D) PFS**** *p* < 0,0001; ** *p* < 0,01; * *p* < 0,05

In order to investigate if miR-335-5p was expressed also in RMS tissues, we used the dataset GSE135518 obtained from the GEO database (https://www.ncbi.nlm.nih.gov/gds/). We observed that miR-335-5p was upregulated in RMS tumor tissues (49 patients) with respect to normal tissues (13 subjects), (*p* = 0.00202), and upregulated significantly between ARMS and ERMS (*p* = 0.04), (Fig. 4A). Furthermore, the miRNA expression correlated with the Intergroup Rhabdomyosarcoma Study (IRS) grouping system, (*p* = 0.0234), (Fig. 4B) and patients with high miR-335-5p expression had a lower Overall- (OS, *p* = 0.044) and Progression Free-survival (PFS, *p* = 0.025), (Fig. 4C, D).

**Fig. 5:**
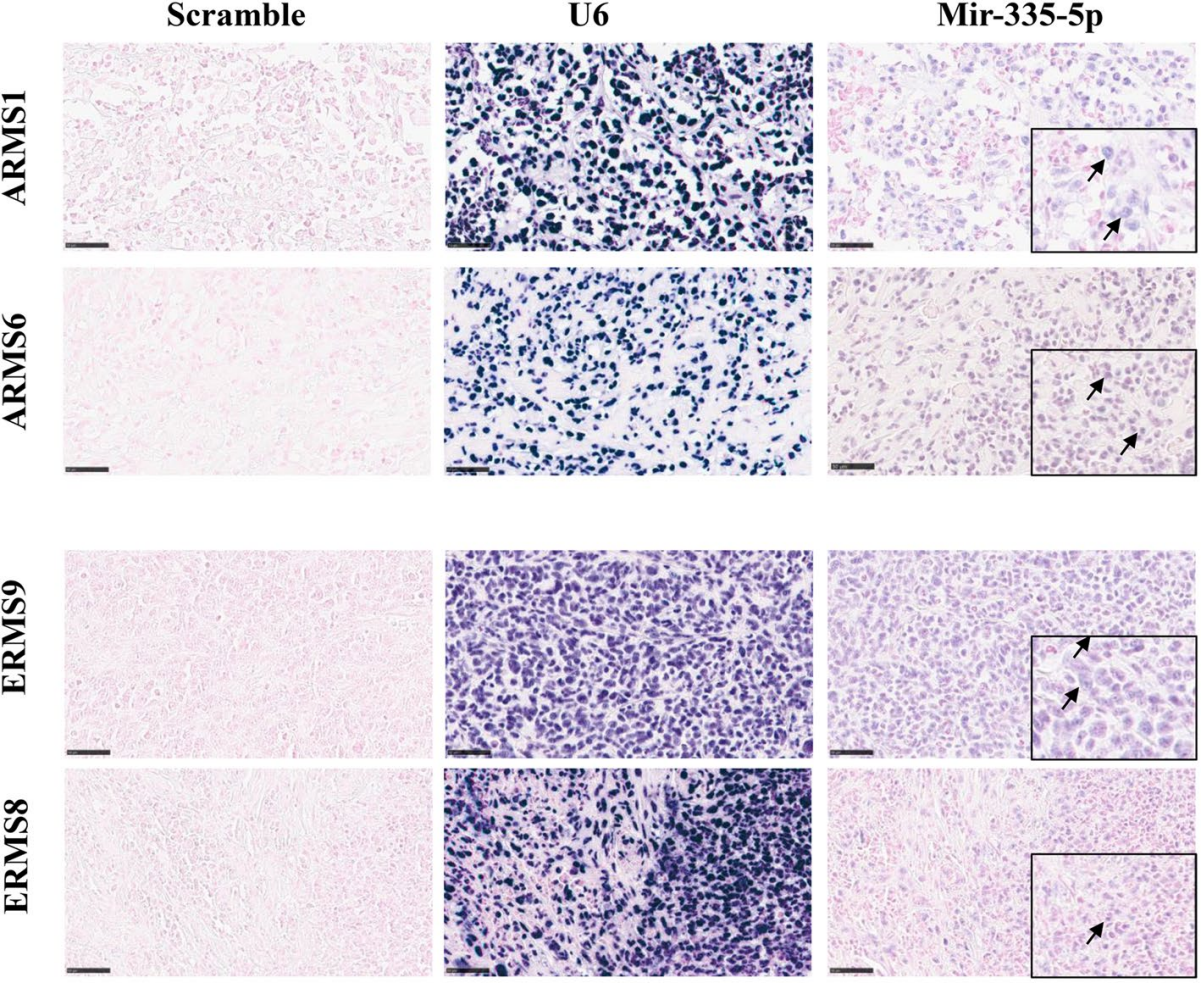
Representative miR-335-5p ISH staining images in tissue samples of RMS patients (× 400). The panel shows the presence of miR-335-5p in tumor cells

To explore miR-335-5p expression in tumor cells and microenvironment, ISH was performed on paraffin-embedded RMS tumor samples of 6 ARMS and 10 ERMS patients. MiR-335-5p signal was observed exclusively in the cytoplasm of cancer cells. With the limitation of low numbers of samples, we observed a median total score of 3.3 in ARMS and 2.3 in ERMS patients (see Materials and Methods section for the score calculation). Representative pictures are shown in Fig. 5.

## Discussion

The use of liquid biopsy to track tumor-related biomarkers at diagnosis, during treatment or follow-up, represents a very promising tool, especially in the field of pediatric tumors, due to the non-invasive characteristics of this approach.

In this study, we performed a miRNA profile of plasma EVs isolated from patients affected by RMS, to identify specific tumor related biomarkers. MiRNAs have been largely studied in RMS tumors; in particular, they can directly modulate myogenic-regulatory factors thereby regulating RMS development and progression. Muscle-specific miRNAs, termed myomiRs, are indeed important for regulation of cell proliferation and myoblasts differentiation, which are key processes for muscle development. The deregulation of myomiRs expression may inhibit the correct skeletal muscle growth causing the occurrence of muscle-related disorders [34–36]. Several myomiRs have been involved in the development of RMS, especially those with tumor suppressive activity.

For example, it has been described that PAX7, which is a gene essential for ARMS cell differentiation and tumor progression, was downregulated by miR-206 [37]. Low miR-206 expression in RMS tissues correlated with poor survival in ERMS and ARMS patients [38]. Among circulating biomarkers, exosomal miRNAs are potentially highly useful since they are stable and have been described to be tumor type-specific [39–41]. Concerning RMS, Ghayad and collaborators reported 31 miRNAs commonly deregulated in exosomes released by ARMS and ERMS cell lines [24]. Interestingly, RMS-derived exosomes increased the migration and invasion of normal recipient fibroblasts and endothelial cells in a dose-dependent manner, underlining their putative contribution to the metastatic process [24]. In a separate work, Hanna and colleagues identified miR-486-5p, a downstream target of the PAX3-FOXO1 chimeric protein, as highly expressed in ARMS cell lines-derived exosomes [18]. In a small cohort of RMS patients (6 ERMS and 1 ARMS), miR-486-5p was enriched in serum exosomes and its value was reduced after chemotherapy [42].

In our first analysis, miR-486-5p was significantly upregulated in ARMS patients in comparison to ERMS and HCs (Table 1), however the validation analysis did not confirm this observation.

We identified miR-335-5p, as significantly upregulated in plasma EVs of patients affected by ARMS compared to HCs and ERMS. The analysis of different time points for two RMS patients demonstrated a reduction of EVs-miR-335-5p expression during treatment, but an increase before diagnosis of disease recurrence. MiR-335-5p expression was increased in ARMS tumor compared to ERMS and surrounding healthy muscle as showed in the GEO dataset. The ISH revealed the localization in the tumor cells. Furthermore miR-335-5p expression correlated with IRS, as well as with OS and PFS.

MiR-335-3p is induced during myoblast differentiation and highly expressed during muscle regeneration [43]. Indeed, deregulation of miR-335 is implicated in several muscle-related diseases [44] being involved in multiple cellular processes including proliferation and apoptosis. In cancer miR‐335 presents a dual role, having both oncosuppressor and oncogenic function depending on tumor type and can affect treatment resistance of cancer [45–48]. Interestingly, exosomes from metastatic colorectal cancer cell lines, carrying miR‐335, promote migration, invasion, and epithelial to mesenchymal transition (EMT) [49]. High levels of miR-335 have been identified in tumors and plasma derived from patients affected by gastric cancer and uterine sarcoma patients with poor prognosis [50, 51]. In this perspective, miR-335 could be considered as a potential prognostic biomarker [52].

Concerning RMS, two teams documented the overexpression of miR-335-5p in RMS tumor samples and in particular in ARMS [53, 54]. Hanna and colleagues observed that silencing of PAX3-FOXO1 in ARMS cell lines induced downregulation of miR-335-3p, while PAX3-FOXO1 overexpression in ERMS cell line determine an upregulation of this miRNA [18].

## Conclusion

In our study we observed that miR-335-5p expression is u*p-*regulated in EVs isolated from plasma and in tumor samples of patients affected by ARMS in comparison to ERMS and HCs and miR335-5p expression appears to be associated with stage and survival in RMS patients. Considering the limited number of samples, it was not possible to define in this study if miR-335-5p could be an independent prognostic biomarker or if the worse prognosis is due to the presence of alveolar histology. Therefore, further studies with larger sample sizes are needed to confirm these findings and to deeply elucidate its biological role.

## Supplementary Information

Supplementary Material 1: Supplementary Methods.

Supplementary Material 2: Supplementary Table 1: Clinical characteristics of the RMS patients involved in the study.  Supplementary Table 2: Clinical characteristics of the RMS patients included in the GEO Dataset (GSE135518).

Supplementary Material 3: Supplementary Figure 1:  Characterization of EVs isolated from plasma of HC and RMS patients. (A) Representative TEM images of plasma EVs isolated from HC, ARMS and ERMS patients. Scale bar = 200 nm. (B) Nanoparticle tracking analysis. The calculated size distribution is depicted as mean (black line) with standard error (red shading). (C) Western blot analysis of the typical EVs proteins, TSG101, CD9, CD63 and endoplasmic reticulum protein Calnexin for representative samples. HSP90 was reported as control. 

Supplementary Material 4: Supplementary Figure 2: Expression of Evs-miR-335-5p at different time point in one ARMS and one ERMS patients. ARMS t1, t2 and t3:  maintenance treatments; t4: local relapse. ERMS t1: diagnosis; t2: 3rd cycle chemotherapy; t3: 4th cycle chemotherapy; t4: local PD.

## Data Availability

All data generated or analyzed during this study are included in this published article. Datasets are described in the material and methods section and public available (accession number is provided).

## Abbreviations

RMS: Rhabdomyosarcoma
ERMS: Embryonal Rhabdomyosarcoma
ARMS: Alveolar Rhabdomyosarcoma
EFS: Event free survival
OS: Overall survival
cfDNA: Circulating free DNA
miRNAs: MicroRNAs
EVs: Extracellular vesicles
EVs-miRNAs: Extracellular Vesicles microRNA
HC: Healthy children
RT: Room temperature
MISEV: Minimal Information for Studies of Extracellular Vesicles
TEM: Transmission Electron Microscopy
TSG101: Tumor Susceptibility Gene 101
BF: Biofluids
FC: Fold change
GEO: Gene Expression Omnibus
CTRL: Normal tissue counterparts
GO: Gene Ontology
ISH: In situ hybridization
IS: Intensity score
PS: Proportional score
PD: Progression disease
IRS: Intergroup Rhabdomyosarcoma Study
EMT: Epithelial to mesenchymal transition
