# Supplementary material for: Plasma-derived extracellular vesicles miR-335–5p as potential diagnostic biomarkers for fusion-positive rhabdomyosarcoma
Source: J Exp Clin Cancer Res. 2024 Oct 9;43:282. doi: 10.1186/s13046-024-03197-3 (PMC11463097; doi:10.1186/s13046-024-03197-3)
Supplement: Supplementary file 1 — Supplementary Material 1: Supplementary Methods. [file 13046_2024_3197_MOESM1_ESM.docx]

**Supplementary Methods**

**Extracellular Vesicles Characterization (NTA, WB, SEM)**

EVs preparations were verified following the recommendations of MISEV2023 [25]. Transmission Electron Microscopy (TEM) was used to verify exosomes preparation. Five ul of each EVs suspension was placed on Parafilm. EVs were allowed to adhere to the surface of Formvar‐coated copper grids (Electron Microscopy Sciences) put on the top of each drop. Grids were contrasted with 2% uranyl acetate after being washed in PBS and distilled water. The observation was performed with a Philips EM208 transmission electron microscope equipped with a digital camera (University Centre of Electron and Fluorescence Microscopy ‐ CUMEF). The NS500 nanoparticle characterization system (NanoSight) equipped with a blue laser (405 nm) was used to characterize exosome size and particle number (CNIO, Madrid). Protein extraction was performed with Cell lysis buffer (Cell Lysis Buffer (10X) #9803 Cell Signalling Technology) containing 10 mM phenylmethylsulphonyl fluoride (PMSF #93482 Sigma) as a protease inhibitor.

Lysates were incubated on ice for 30 min and centrifugated at 12 000 × g for 20 min at 4°C. Equal micrograms (10 μg) of proteins quantified with bicinchoninic acid (BCA) assay (Thermo Scientific) and boiled in SDS sample buffer (4x Laemmli Sample Buffer BIORAD cat. #161-0747) were resolved on 10% SDS-PAGE and transferred to PVDF membranes (Immobilon®-P Transfer Membrane Merck Millipore cat. IPVH00010). Blots were blocked for 1 h in PBS-T (PBS plus 0.05% Tween-20), 5% non-fat, dried milk and probed overnight at 4°C with anti-TSG101 (4A10) ab83 (Abcam), anti-CD9 (C-4) sc-13118 (Santa Cruz Biotechnology), CD63 (H-193): sc-15363 (Santa Cruz Biotechnology), anti-CALNEXIN (E-10) sc-46669 (Santa Cruz Biotechnology), and anti-HSP90α/β (F-8) sc-13119 (Santa Cruz Biotechnology). Immunocomplexes were detected with horseradish peroxidase-conjugated species-specific secondary antibodies (Santa Cruz Biotechnology) followed by enhanced chemiluminescence reaction with Immobilon Western Chemiluminescence HRP substrate WBKLS0100 (Millipore).
