# Supplementary material for: Plasma-derived extracellular vesicles miR-335–5p as potential diagnostic biomarkers for fusion-positive rhabdomyosarcoma
Source: J Exp Clin Cancer Res. 2024 Oct 9;43:282. doi: 10.1186/s13046-024-03197-3 (PMC11463097; doi:10.1186/s13046-024-03197-3)
Supplement: Supplementary file 2 — Supplementary Material 2: Supplementary Table 1: Clinical characteristics of the RMS patients involved in the study. Supplementary Table 2: Clinical characteristics of the RMS patients included in the GEO Dataset (GSE135518). [file 13046_2024_3197_MOESM2_ESM.docx]

**SUPPLEMENTARY TABLES**

**Supplementary Table 1**: Clinical characteristics of the RMS patients involved in the study

| Pt | Age  (mos) | Gender | Histology | Primary site | Primary size  (cm) | Metastasis | Nodes | IRSa  Group |
| --- | --- | --- | --- | --- | --- | --- | --- | --- |
| ERMS1 | 188 | M | ERMS | Extremity | >5 | none | N0 | III |
| ERMS2 | 105 | F | ERMS | HN PM | >5 | none | N1 | III |
| ERMS3 | 46 | F | ERMS | orbit | ≤5 | none | N0 | III |
| ERMS4 | 52 | M | ERMS | GU BP | > 5 | none | N0 | III |
| ERMS5 | 73 | M | ERMS | HN PM | >5 | none | N0 | III |
| ERMS6 | 63 | M | ERMS | Lung metastasis | NA | NA | NA | NA |
| ERMS7 | 126 | M | ERMS | HM non PM | > 5 | Lung/Bone | N1 | IV |
| ERMS8 | 17 | F | ERMS | Extremity | >5 | none | N0 | III |
| ERMS9 | 5 | M | ERMS | Abdomen | >5 | Lung | N0 | IV |
| ERMS10 | 45 | M | ERMS | HN PM | ≤5 | none | N1 | III |
| ERMS11 | 48 | M | ERMS | HN PM | <5 | Lung/Bone | N0 | IV |
| ERMS12 | 5 | F | ERMS | perineum | >5 | none | N0 | III |
| ARMS1 | 10 | M | ARMS | HN PM | ≤5 | Bone/BM/Nodes | N1 | IV |
| ARMS2 | 181 | F | ARMS | Chest wall |  | none | N0 | III |
| ARMS3 | 177 | F | ARMS | PELVIS | >5 | Bone | N1 | IV |
| ARMS4 | 5 | F | ARMS | HN PM | >5 | Abdomen, skin, brain (parenchyma and liquor) | N1 | IV |
| ARMS5 | 49 | F | ARMS | HN PM | ≤5 | none | N1 | III |
| ARMS6 | 174 | M | ARMS | HN PM | ≤5 | none | N1 | III |
| ARMS7 | 178 | M | ARMS | HN PM | ≤5 | none | N1 | III |
| ARMS8 | 47 | M | ARMS | Abdomen | >5 | Bone/ BM | N0 | IV |
| ARMS9 | 111 | M | ARMS | Extemity | >5 | none | N1 | III |
| HC1 | 55 | M | NA | NA | NA | NA | NA | NA |
| HC2 | 61 | F | NA | NA | NA | NA | NA | NA |
| HC3 | 35 | M | NA | NA | NA | NA | NA | NA |
| HC4 | 18 | M | NA | NA | NA | NA | NA | NA |
| HC5 | 14 | M | NA | NA | NA | NA | NA | NA |
| HC6 | 119 | F | NA | NA | NA | NA | NA | NA |
| HC7 | 207 | M | NA | NA | NA | NA | NA | NA |
| HC8 | 6 | M | NA | NA | NA | NA | NA | NA |
| HC9 | 69 | M | NA | NA | NA | NA | NA | NA |
| HC10 | 23 | F | NA | NA | NA | NA | NA | NA |
| HC11 | 44 | F | NA | NA | NA | NA | NA | NA |
| HC12 | 60 | M | NA | NA | NA | NA | NA | NA |
| HC13 | 140 | F | NA | NA | NA | NA | NA | NA |

Pt: patient, mos: months, M: male, F: female, ARMS: alveolar rhabdomyosarcoma, ERMS: embryonal rhabdomyosarcoma, HC: Healthy Children, HN non PM: head and neck non-parameningeal, GU BP: genitourinary bladder or prostate, HN PM: Head and neck parameningeal, BM :bone-marrow, N0: no clinical or pathological node involvement, N1: clinical or pathological nodal involvement, NA: Not Applicable, IRSa: post-surgical stage according to Intergroup Rhabdomyosarcoma Study (IRS) grouping system [55].

**Supplementary Table 2:** Clinical characteristics of the RMS patients included in the GEO Dataset (GSE135518).

| Sample geo accession | Age (mos) | Gender | Histology | IRS | OS (mos) | Survival data |
| --- | --- | --- | --- | --- | --- | --- |
| GSM4013549 | 174.81 | F | ARMS | 4 | 9 | DOD |
| GSM4013557 | 274.45 | F | ARMS | 3 | 20 | DOD |
| GSM4013558 | 20.3178 | F | ARMS | 3 | 64 | 2CR |
| GSM4013562 | 376.04 | M | ARMS | 4 | 10 | 1CR |
| GSM4013564 | 332.81 | M | ARMS | 4 | 43 | DOD |
| GSM4013568 | 95.6712 | F | ARMS | 3 | 209 | 2CR |
| GSM4013570 | 83.8685 | M | ARMS | 3 | 140 | 1CR |
| GSM4013571 | 227.18 | F | ARMS | 3 | 10 | DOD |
| GSM4013573 | 192.62 | F | ARMS | 3 | 22 | DOD |
| GSM4013576 | 318.9 | F | ARMS | 1 | 107 | 1CR |
| GSM4013580 | 16.9644 | F | ARMS | 3 | 147 | 1CR/DOC |
| GSM4013581 | 170.47 | F | ARMS | 4 | 16 | DOD |
| GSM4013582 | 269.03 | M | ARMS | 3 | 11 | DOD |
| GSM4013584 | 249.47 | M | ARMS | 4 | 9 | DOD |
| GSM4013590 | 411.95 | M | ARMS | 4 | 14 | DOD |
| GSM4013591 | 155.31 | M | ARMS | 4 | 13 | DOD |
| GSM4013599 | 253.12 | M | ARMS | 4 | 11 | 1CR |
| GSM4013605 | 228.43 | F | ARMS | 4 | 23 | DOD |
| GSM4013609 | 83.5397 | F | ARMS | 1 | 99 | 1CR |
| GSM4013610 | 266.89 | F | ARMS | NA | NA | NA |
| GSM4013550 | 187.07 | M | ERMS | 2 | 71 | 1CR |
| GSM4013551 | 31.2657 | M | ERMS | 3 | 283 | 1CR |
| GSM4013553 | 17.6219 | M | ERMS | 3 | 68 | 1CR |
| GSM4013554 | 198.87 | M | ERMS | 1 | 117 | 1CR |
| GSM4013559 | 24.6247 | F | ERMS | 3 | 57 | 1CR |
| GSM4013560 | 270.77 | M | ERMS | 2 | 87 | 1CR |
| GSM4013561 | 35.6055 | F | ERMS | 3 | 67 | 1CR |
| GSM4013567 | 285.21 | M | ERMS | 4 | 5 | DOD |
| GSM4013569 | 124.31 | M | ERMS | 3 | 31 | 1CR |
| GSM4013572 | 47.5397 | M | ERMS | 3 | 15 | DOC |
| GSM4013574 | 60.4603 | M | ERMS | 1 | 313 | 1CR |
| GSM4013575 | 261.27 | M | ERMS | 3 | 17 | DOD |
| GSM4013577 | 18.8384 | M | ERMS | 3 | 8 | DOD |
| GSM4013578 | 50.6301 | M | ERMS | 3 | 285 | 1CR |
| GSM4013583 | 167.21 | F | ERMS | 2 | 73 | 1CR |
| GSM4013586 | 38.8932 | F | ERMS | 3 | 17 | DOD |
| GSM4013588 | 43.2 | F | ERMS | 3 | 74 | 1CR |
| GSM4013589 | 159.12 | F | ERMS | 3 | 22 | DOD |
| GSM4013592 | 176.58 | F | ERMS | 4 | 23 | DOD |
| GSM4013593 | 184.41 | F | ERMS | 1 | 28 | 1CR |
| GSM4013595 | 192.07 | M | ERMS | 1 | 177 | 1CR |
| GSM4013596 | 176.09 | F | ERMS | 3 | 32 | 2CR |
| GSM4013597 | 407.97 | F | ERMS | 3 | 25 | 1CR |
| GSM4013600 | 47.8027 | F | ERMS | 4 | 26 | DOD |
| GSM4013601 | 144.03 | M | ERMS | 1 | 36 | DOD |
| GSM4013602 | 99.9452 | M | ERMS | 3 | 240 | 1CR |
| GSM4013604 | 162.54 | M | ERMS | 1 | 120 | 1CR |
| GSM4013606 | 159.91 | M | ERMS | 1 | 175 | 1CR |
| GSM4013612 | 382.65 | M | ERMS | NA | NA | NA |
| GSM4013552 | 192.07 | M | adjacent_normal | 1 | NA | NA |
| GSM4013556 | 60.4603 | M | adjacent_normal | 1 | NA | NA |
| GSM4013563 | 228.43 | F | adjacent_normal | 4 | NA | NA |
| GSM4013565 | 95.6712 | F | adjacent_normal | 3 | NA | NA |
| GSM4013566 | 72.7233 | M | adjacent_normal | 4 | NA | NA |
| GSM4013579 | 198.87 | M | adjacent_normal | 1 | NA | NA |
| GSM4013585 | 83.8685 | M | adjacent_normal | 3 | NA | NA |
| GSM4013587 | 192.62 | F | adjacent_normal | 3 | NA | NA |
| GSM4013598 | 270.77 | M | adjacent_normal | 2 | NA | NA |
| GSM4013607 | 382.65 | M | adjacent_normal | NA | NA | NA |
| GSM4013608 | 83.5397 | F | adjacent_normal | 1 | NA | NA |
| GSM4013555 | 83.8685 | M | normal | 3 | NA | NA |
| GSM4013594 | 17.6219 | M | normal | 3 | NA | NA |
| GSM4013603 | 95.6712 | F | normal | 3 | NA | NA |
| GSM4013611 | 144.03 | M | normal | 1 | NA | NA |

*mos:* months, *M:* male, *F:* female, *ARMS:* alveolar rhabdomyosarcoma, *ERMS:* embryonal rhabdomyosarcoma, *NA:* Not Applicable, IRSa: post-surgical stage according to Intergroup Rhabdomyosarcoma Study (IRS) grouping system [55]; 1◦ CR: the complete disappearance of disease after first line treatment; 2◦ CR: the complete disappearance of disease after second line therapy, following tumor relapse; DOD: Died of disease; DOC: Died of other causes.
