# Supplementary figures and images for: Plasma-derived extracellular vesicles miR-335–5p as potential diagnostic biomarkers for fusion-positive rhabdomyosarcoma
Source: J Exp Clin Cancer Res. 2024 Oct 9;43:282. doi: 10.1186/s13046-024-03197-3 (PMC11463097; doi:10.1186/s13046-024-03197-3)

# Supplementary Figure 1

A

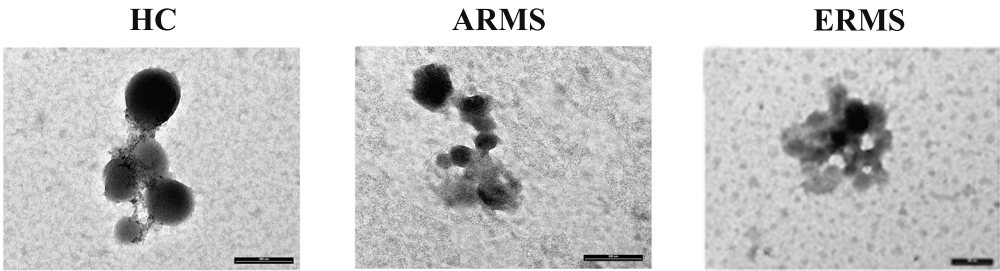

B

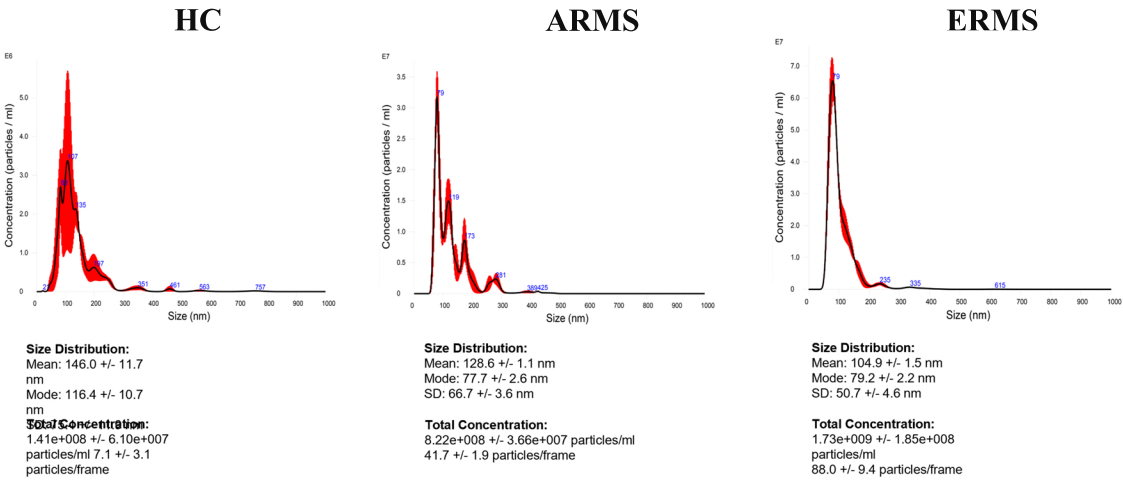

C

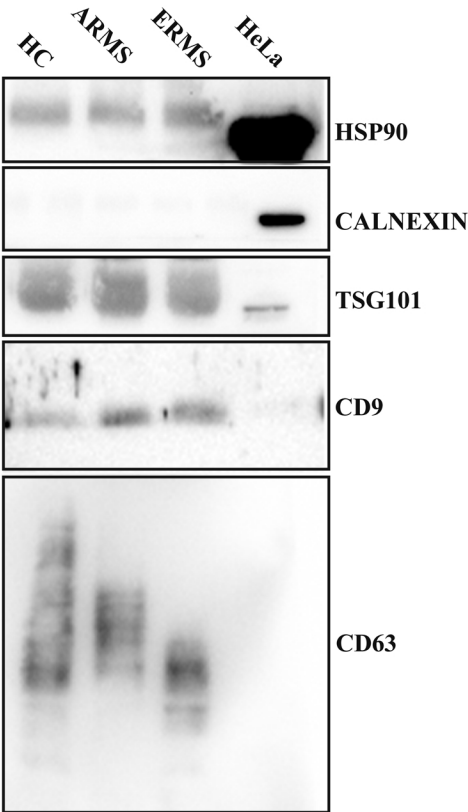

Supplement: Supplementary file 3 — Supplementary Material 3: Supplementary Figure 1: Characterization of EVs isolated from plasma of HC and RMS patients. (A) Representative TEM images of plasma EVs isolated from HC, ARMS and ERMS patients. Scale bar = 200 nm. (B) Nanoparticle tracking analysis. The calculated size distribution is depicted as mean (black line) with standard error (red shading). (C) Western blot analysis of the typical EVs proteins, TSG101, CD9, CD63 and endoplasmic reticulum protein Calnexin for representative samples. HSP90 was reported as control. [file 13046_2024_3197_MOESM3_ESM.pdf]

Supplementary Figure 2

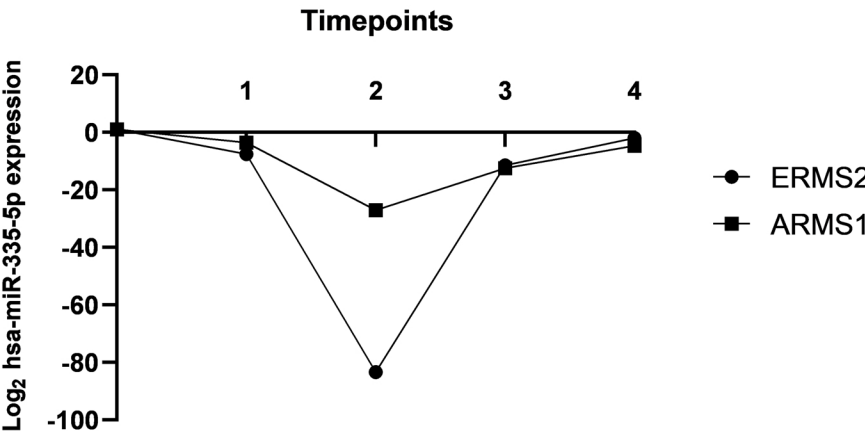

Supplement: Supplementary file 4 — Supplementary Material 4: Supplementary Figure 2: Expression of Evs-miR-335-5p at different time point in one ARMS and one ERMS patients. ARMS t1, t2 and t3: maintenance treatments; t4: local relapse. ERMS t1: diagnosis; t2: 3rd cycle chemotherapy; t3: 4th cycle chemotherapy; t4: local PD. [file 13046_2024_3197_MOESM4_ESM.pdf]
